# Inflammation in Metabolic and Cardiovascular Disorders—Role of Oxidative Stress

**DOI:** 10.3390/life11070672

**Published:** 2021-07-09

**Authors:** Ying Sun, Elias Rawish, Henry M. Nording, Harald F. Langer

**Affiliations:** 1 Cardioimmunology Group, Medical Clinic II, University Heart Center Lübeck, 23538 Lübeck, Germany; ying.sun@student.uni-luebeck.de (Y.S.); elias.rawish@uksh.de (E.R.); henry.nording@uksh.de (H.M.N.); 2 University Heart Center Lübeck, Medical Clinic II, University Hospital, 23538 Lübeck, Germany; 3 DZHK (German Research Centre for Cardiovascular Research), Partner Site Hamburg/Lübeck/Kiel, 23562 Lübeck, Germany

**Keywords:** oxidative stress, cardiovascular diseases, metabolic disorders, adipose tissue, hypothalamic dysfunction

## Abstract

Cardiovascular diseases (CVD) constitute the main cause of death worldwide. Both inflammation and oxidative stress have been reported to be involved in the progress of CVD. It is well known that generation of oxidative stress during the course of CVD is involved in tissue damage and inflammation, causing deleterious effects such as hypertension, dysfunctional metabolism, endothelial dysfunction, stroke, and myocardial infarction. Remarkably, natural antioxidant strategies have been increasingly discovered and are subject to current scientific investigations. Here, we addressed the activation of immune cells in the context of ROS production, as well as how their interaction with other cellular players and further (immune) mediators contribute to metabolic and cardiovascular disorders. We also highlight how a dysregulated complement system contributes to immune imbalance and tissue damage in the context of increases oxidative stress. Additionally, modulation of hypothalamic oxidative stress is discussed, which may offer novel treatment strategies for type-2 diabetes and obesity. Together, we provide new perspectives on therapy strategies for CVD caused by oxidative stress, with a focus on oxidative stress.

## 1. Introduction

Several cardiovascular diseases (CVD), including atherosclerosis, myocardial infarction (MI), and heart failure, are related to low-grade inflammation. A balanced interaction between ongoing immune responses and metabolic regulation were demonstrated to be decisive homeostatic mechanism, as dysregulation of this balance may lead to various chronic metabolic disorders, particularly obesity, type 2 diabetes, and subsequent CVD [1]. Different players of inflammation such as several types of immune cells and mediators of the innate immune system are major contributors to the pathogenesis of these conditions. Furthermore, oxidative stress has been reported as a driver of metabolic and CVD: An excessive generation of reactive oxygen species (ROS) can tilt the equilibrium between protective and harmful effects, resulting in oxidative stress and subsequent release of proinflammatory cytokines, endothelial dysfunction, and reduction of nitrogen monoxide (NO) utilization. Thus, the present review describes and summarizes the miscellaneous principles of inflammation contributing to metabolic disorders and CVD, which are associated with oxidative stress.

## 2. Innate Immune Activation Contributes to Metabolic Disorders

### 2.1. Macrophages, Dendritic Cells, and Further Immune Cells Contributing to Inflammation in Metabolic Disorders

To understand the intersection between metabolism and inflammation, one should consider the functional links of cells featuring metabolic or immune properties, such as macrophages, dendritic cells (DCs), and adipocytes [1]. Macrophages and DCs provide an early defense barrier against external pathogens [2]. In addition, both cell types respond to “danger signals” and cytokines, such as metabolic reprogramming in response to hypoxia or nutrient alterations [3]. Two different inflammatory phenotypes are known for macrophages: the M1-like macrophages are activated classically, typically by interferon (IFN)-γ or lipopolysaccharides (LPS), while M2-like macrophages are activated alternatively by exposure to several cytokines such as interleukin (IL)-4, IL-10, or IL-13. M1 macrophages secrete proinflammatory factors such as tumor necrosis factor-α (TNF-α), IL1β, and inducible nitric oxide synthase (iNOS)/NO [2,3,4,5,6]. By contrast, M2 macrophages have been linked to diminished levels of NO and TNF-α, as well as the generation of anti-inflammatory cytokines such as IL-10 [2]. During obesity, M1-like macrophage numbers elevate due to augmented levels of free fatty acids (FFAs), cholesterol, LPS, and hypoxia [7], contributing to adipose tissue (AT) inflammation and inhibition of insulin signaling. In contrast, anti-inflammatory M2-like macrophages dominate in lean humans and mice [8].

DCs are antigen-presenting cells of the mammalian immune system. They present internalized antigen material to T cells of the immune system. Thereby, they constitute an intersection point between the innate and the adaptive immune system [9]. DCs can be divided into plasmacytoid DCs (pDCs) and conventional DCs (cDCs), distinguished by morphology and function. Resting DCs have been demonstrated to display a catabolic metabolism, steadily decomposing nutrients for energy generation [10]. Thereby, tricarboxylic acid (TCA) cycle fueled via fatty acid β-oxidation (FAO) and glutaminolysis, drive activation of oxidative phosphorylation (OXPHOS), which is regulated by AMP-activated protein kinase (AMPK) [2,3,11,12,13]. Besides glucose, intracellular glycogen is used by resting DCs to support basal glycolytic demands, driving mitochondrial respiration [14]. Mature DCs perceive and respond to environmental stimuli. In secondary lymphoid tissues, DCs present antigens to T cells, promoting T cell proliferation as well as differentiation. Mice deficient of Flt3l (Fms-related tyrosine kinase 3 ligand) show a lack of DCs as well as reduced amounts of natural killer cells and regulatory T cells and B cells, while insulin sensitivity in diet-induced obesity is improved. This finding points out the pivotal role of DCs in the regulation of systemic metabolism [15]. Accordingly, CD11^+^DCs were identified in AT during insulin resistance inducing differentiation of Th17 in metabolic processes [16,17]. Interestingly, DCs have recently been shown to promote hypertension by mediating fluid retention and renal oxidative stress [18].

Eosinophils are cells of the innate immune system [19]. It is known that ROS can induce the death of eosinophils. Siglec-8 connection has been shown to enhance IL-5 in a ROS-dependent manner, inducing ERK phosphorylation, leading to eosinophil death [20]. Importantly, a synergistic effect of type 2 innate lymphoid cells (ILC2), eosinophils, and M2 macrophages controls metabolic homeostasis in visceral adipose tissue (VAT). Increased interaction of ILC2 with IL-33 in VAT leads to accumulation of eosinophils and goes together with a reduction of eosinophils in the bone marrow and spleen [21]. However, eosinophils are also related to the development of CVD [22]. Overexpression of eosinophil-specific chemokines, such as eotaxin, in smooth muscle cells of vessels have been demonstrated to stimulate TNF-α, interferon-α, and interferon-β production, promoting atherosclerosis, by regulating macrophages to stimulate the formation of foam cells [23,24,25].

### 2.2. Different Types of Adipose Tissue

**Figure 1:**
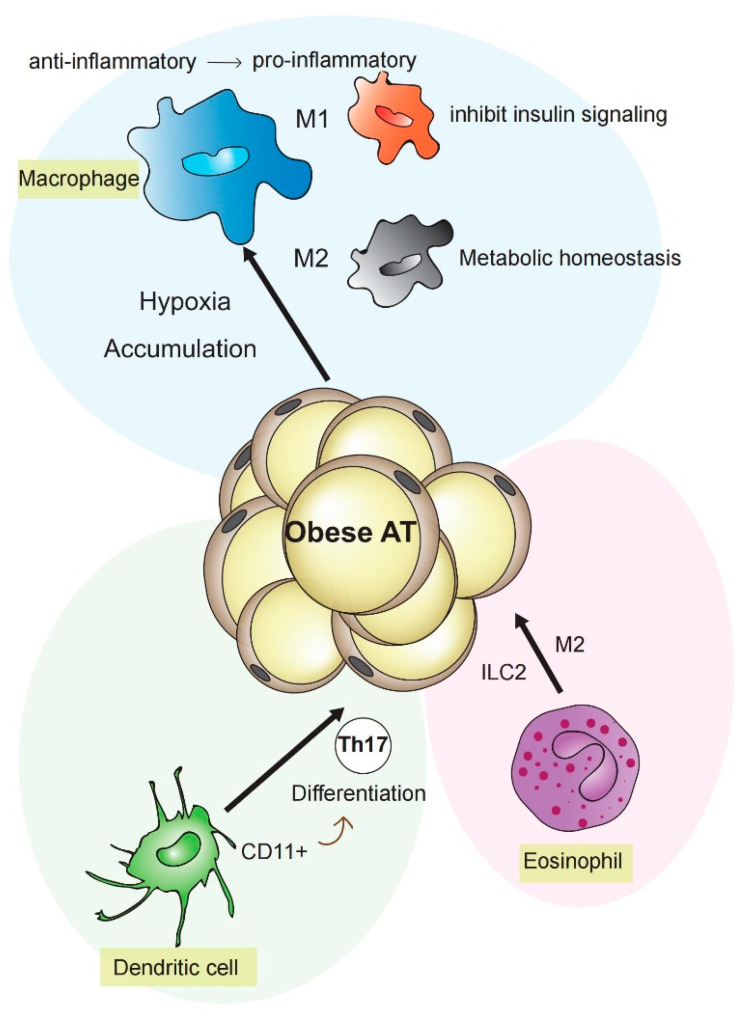
Contribution of different cell types to inflammation in adipose tissue. Mature CD11^+^DCs induce differentiation of Th17 under obesity-related insulin resistance and contribute to adipose tissue inflammation. Eosinophils, M2 macrophages, and type 2 innate lymphoid cells regulate metabolic homeostasis in adipose tissue by cytokine expression or dependence upon the γc cytokine chain. Under obesity conditions, M1-like macrophages lead to insulin resistance. Following the adipose increase, macrophage phenotype changed from anti-inflammatory M2 to pro-inflammatory M1 type.

AT is classically regarded as a tissue that stores surplus nutrients. During the last two decades of research, it has been found that AT is also a key regulator of body metabolism and homeostasis, as it secretes a variety of adipokines [26] (Figure 1). Subcutaneous and visceral adipose tissues display hyperplasia and hypertrophy following nutritional overload, resulting in adipokine dysregulation, hypoxia, and subsequent low-grade inflammation, which in turn fuels the infiltration and activation of immune cells into AT [27].

There are two main types of AT: WAT which consists of unilocular adipocytes that store energy and regulate metabolic homeostasis by the secretion of adipokines, and brown adipose tissue (BAT), which is shaped of mitochondria-rich multilocular adipocytes whose prior function is energy dissipation by thermogenesis [28]. Accumulation of WAT cells, local intrusion of immune cells, and increased levels of pro-inflammatory cytokines lead to peripheral insulin resistance in obesity [29]. In comparison to WAT, BAT of high-fat diet (HFD)-treated mice has shown a lower of immune cell-enriched mRNA expression and macrophage infiltration, indicating that BAT “resists” obesity-induced inflammation [30]. However, similar to WAT, BAT from mice with a sufficiently sustained obesogenic diet finally exhibited high mRNA levels of inflammation markers, such as TNF-α [31,32].

BAT is enriched by multilocular lipid droplets, high vascularization, and abundant mitochondria. As mentioned, BAT severs pivotally in thermoregulation through lipid oxidation-mediated heat generation [33]. Evidence suggests that the majority of WAT depots can switch phenotypically to brown fat under particular conditions, such as bariatric surgery, severe burns, cancer cachexia, cold exposure, and drug ingredients [34,35,36,37,38,39,40].

### 2.3. Platelets, Diabetes, and Its Sequel

Oxidative stress is of decisive importance for the development and maintenance of microvascular and macrovascular diabetes complications [41]. Under the metabolic environment of type 2 diabetes mellitus (T2DM), activated platelets can release chemokines such as CXCL4, CXCL5, and CCL5 on vascular cell surfaces, triggering atherogenesis [42,43,44,45]. Furthermore, several platelet receptors, e.g., P-Selectin, P2Y1 receptor, protease-activated receptors (PARs), and glycoprotein Ib-IX-V complex mediate the formation and activation of platelet–leukocyte microparticle complexes, fueling inflammation and subsequent atherosclerosis [46,47]. Due to different antioxidant enzymes, ROS expression is kept low in healthy patients. However, diabetic patients’ platelets display a reduced antioxidant capacity, and hence they are unable to adequately scavenge ROS [48], which in turn causes altered platelet function, dysregulated calcium homeostasis, and activation of protein kinase C (PKC). PKC acts as a mediator to promote platelet aggregation [49,50,51]. In particular, platelet CD40 ligand (CD40L) interacts with endothelial CD40, which fuels the production of chemokines, such as monocyte chemotactic protein 1 (MCP1) and IL-8, but also the expression of adhesion molecules such as vascular cell adhesion molecule 1 (VCAM-1) and E-selectin, which initiates an inflammatory response at the vessel wall. Furthermore, soluble CD40L (sCD40L) can be secreted by activated platelets express, inducing endothelial surface expression of P-selectin and secretion of IL-6 [52]. Interestingly, sCD40L mediates stimulation-induced platelet release of ROS and nitrogen species by activation of mitogen activated protein kinase (MAPK) and Akt signaling pathway, further amplifying platelet activation and indicating a crucial role of sCD40L and oxidative stress in mediating platelet-dependent inflammatory and thrombotic responses [53].

In vitro studies have shown several functional abnormalities in platelets of diabetes patients, such as hypersensitivity of platelets to aggregants and hyposensitivity of antiaggregants, indicating promotion of atherosclerosis by increasing platelet activity at site of vascular injury [54]. In diabetes patients, the prothrombotic tendency and platelet reactivity is accompanied by excessive oxidative stress and increased lipid peroxidation due to enhanced free radical activity [55,56]. Overall, platelet dysfunction leads to expanding risk of CVD in patients with T2DM.

### 2.4. Platelets and the Fat Tissue

In the process of adipose tissue hyperplasia, the release of certain adipokines such as IL-6, TNF-α, leptin, and resistin is increased, leading to endothelial dysfunction, followed by increase of vasoconstrictors, expression of adhesion factors, and decrease of vasodilator molecules [57,58,59]. The reduction of vasodilator molecules contributes to prothrombotic conditions, including increased concentrations of plasminogen activator inhibitor 1 and fibrinogen, leading to platelet activation/aggregation. This interaction between AT and platelets is of importance for the regulation of metabolic pathways.

Tozawa et al. [60] created an adipose-derived mesenchymal stem/stromal cell line (ASCL) that was cultured in megakaryocyte induction media. ASCL differentiated into megakaryocytes after 8 days, and platelets (ASCL-PLTs) were released after an additional 4 days. ASCL-PLTs resembled peripheral blood platelets and may have a further function as mesenchymal stem cells (MSCs). ASCL-PLTs do not need gene transfer or exogenous growth factors and display the same in vivo kinetics after application into irradiated immunodeficient mice.

## 3. Oxidative Stress as a Mediator of Tissue Damage and Promotor of Inflammation

The imbalance between the formation of ROS and ROS-degrading antioxidant systems can lead to CVD. This imbalance results in reduced endothelial nitric oxide synthase (eNOS), enhanced mitochondrial superoxide bioavailability, and subsequent exacerbation of hypertension [61]. Overproduction of ROS leads to NO degradation, and uncoupling of eNOS also contributes to a reduction in NO production and increased release of RNS. This mechanism is a crucial mediator of endothelial dysfunction and increases the occurrence of coronary artery disease (CAD) [62].

Nicotinamide adenine dinucleotide phosphate (NADPH) oxidase is a well-known source of superoxide in vascular disease and an important mediator of oxidative stress. Interestingly, McCann et al. [63] have shown that endothelin-1-induced stroke in rat is associated to activation of two catalytic subunits of NADPH, Nox2, and Nox4. Blockade of these subunits might be helpful for treatment of stroke associated brain injury.

NO is an important vasodilator that has a protective effect on blood vessels. Oxidative stress and inflammation are the main driving forces of endothelial dysfunction. Several oxidase systems such as NADPH oxidase, xanthine oxidase, cyclooxygenase, lipoxygenase, myeloperoxidase, cytochrome P450 monooxygenase, uncoupled NOS, and peroxidase can cause NO inactivation, which represents an important mechanism that leads to endothelial dysfunction by increasing the level of superoxide dismutase [64,65]. Under a long-term oxidative stress environment, with accumulation of ROS, cell structures and functions may be damaged, inducing somatic mutations and tumorigenic transformation in several tissues [66,67]. For instance, increased ROS in prostate may cause somatic DNA mutations, promoting genetic instability, cell cycle arrest, and senescence, consequently causing the development of prostate cancer [67].

## 4. Complement Activation and Oxidative Stress

**Figure 2:**
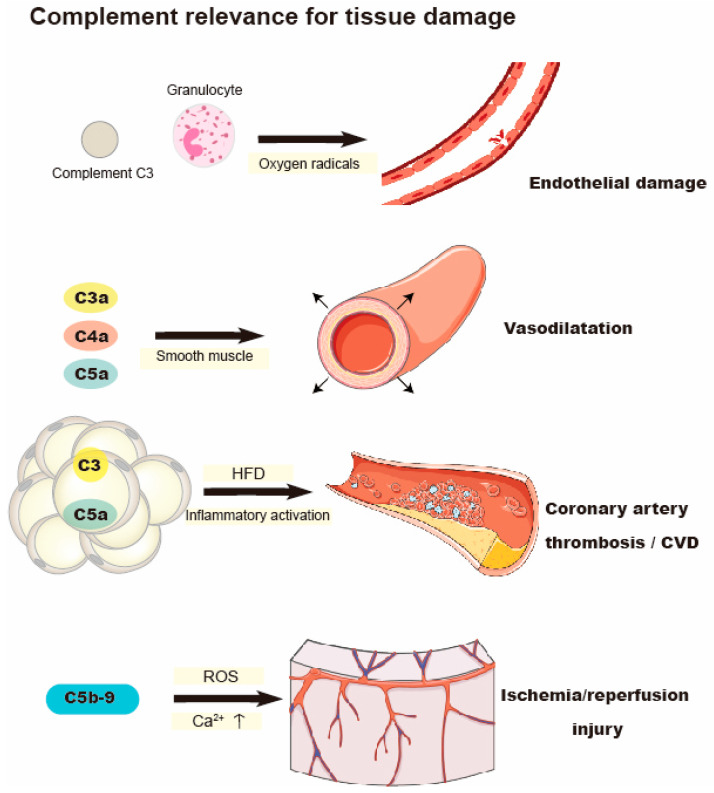
Complement-mediated oxidative stress and its relevance to tissue damage. After exposure to complement C3, oxygen radicals produced by granulocytes contribute to the endothelial damage. C3, C3a, C4a, and C5a are involved in vessel damage in CVD. Activated C5b-9 aggravates ischemia/reperfusion injury.

The complement system is an imperative part of the innate immune system. Excessive activation of host cells or inadequate control of complement activation can lead to immune dysregulation, which intensifies the vicious circle between complement, inflammatory cells, and tissue damage, and aggravates diverse clinical complications [68] (Figure 2). When granulocytes are exposed to activated complement (C), endothelial damage is induced. This damage is mainly mediated by oxygen radicals generated by granulocytes [69]. The indirect effects of anaphylatoxin and the direct effects of membrane attack complex C5b-9 can regulate white blood cell response, change vascular homeostasis, and lead to cell activation and early myocardial ischemia/reperfusion injury [70]. Activated C5b-9 modifies the production of ROS and Ca^2+^ Flux, aggravating ischemia/reperfusion injury [71]. Following ischemia-reperfusion injury, inhibition of C5b-9 reduces the expression of inflammatory factors [72]. In addition, overexpression of C5a can accelerate the development of atherosclerosis in ApoE^−/−^ mice due to increasement of inflammatory activation, macrophage recruitment, and foam cell formation [73]. Beyond C5b-9, C3a, C4a, and C5a act on smooth muscle to promote vasodilatation [74,75]. Furthermore, C5a can promote neutrophil aggregation, chemotaxis, formation of ROS, and arachidonic acid metabolites. C5a regulates the adhesion of neutrophils to the endothelium by release of platelet activating factor [76,77]. Levels of C3 can predict prognosis of heart failure and is negatively correlated with cardiac remodeling [78]. Notably, C3 has been indicated as an important marker of insulin resistance in aged population [79]. AT can activate the alternative complement pathway in T2DM, which causes a low-grade inflammation [80]. The receptors of C3a (C3aR) and C5a (C5aR1 and C5aR2) have been reported to be expressed in adipocytes [81]. Therefore, in addition to the production of complement components, obesity is also a potential target of complement action. After HFD feeding, the expression of C3aR in WAT is increased. Both macrophages and WAT express large amounts of C3aR [82]. Onat et al. found that the increased level of complement C3 is related to the enhanced likelihood of CAD [83]. Furthermore, Complement C3 and C3a have separate roles in pathways leading to CVD. C3a was independently associated with aggravate atherosclerosis in heavy smokers and further promote CVD. On the contrary, in heavy smokers, C3 was exclusively associated with atherothrombosis rather than atherosclerosis [81].

## 5. Oxidative Stress Causes Hypothalamic Dysfunction in Metabolic Disease

There is growing evidence that obesity causes pervasive changes to the energy balance centers of the hypothalamus, perpetuating hyperphagia and metabolic disbalance. Mechanistically, HFD has been shown to promote an accumulation of toxic lipid species, thereby inducing an elevation of markers of inflammation, endoplasmic reticulum (ER) stress, and oxidative stress, which in turn leads to the loss of central leptin sensitivity, loss of insulin sensitivity, and promotion of subsequent obesity [84]. Interestingly, Kjaergaard et al. have investigated the effects of maternal intake of chocolate and soft drink (S) on hypothalamic homeostasis in rats. It has been demonstrated that hypothalamic oxidative stress is detectable prior to the inflammatory response in offspring exposed to maternal S. Furthermore, both maternal and postnatal S promoted hypothalamic inflammation prior to weight gain and diminished peripheral glucose homeostasis, pointing to a causative mechanism [85]. Indeed, hypothalamic inflammation and oxidative stress have been demonstrated to begin as early as after 1 week of HFD treatment [86]. Excessive levels of ROS were shown to alter various cellular components such as DNA, proteins, and lipids, leading to neuronal damage, thus activating several cellular inflammatory pathways [86]. In particular, neuronal oxidative stress may promote the activation of the c-Jun N-terminal kinase (JNK) and nuclear factor κB (NFκB) pathways [1,87], which are well known to induce suppressor of cytokine signaling 3 (SOCS3) and PTP1B (protein tyrosine phosphatase 1B) expression, thereby promoting central leptin resistance by inhibition of signal transducer and activator of transcription 3 (STAT3) phosphorylation as a part of the leptin post-receptor signaling [88]. Fascinatingly, the anorectic drug phenylpropanolamine has been able to restore hypothalamic STAT3 phosphorylation and decrease body weight by increasing levels of antioxidants [89]. In accordance, several beneficial effects on metabolic syndrome have been attributed to polyphenols, in part via modulation of hypothalamic inflammation and oxidative stress [90]. Thus, these insights could point to a novel new direction to research on hypothalamic energy regulation by considering the mechanisms by which oxidative stress regulates energy homeostasis, which may offer novel treatment strategies for obesity and T2DM.

## 6. Translational Implications

To develop novel therapy strategies for infections and cardiovascular diseases caused by oxidative stress, antioxidant therapy, reducing the ROS generation system and improving the antioxidant mechanism, receives major consideration. New ROS scavengers that target mitochondrial ROS are currently being studied, for example, mitochondrial-targeted antioxidant mitoquinone. It reduces the formation of free radicals without affecting the oxidative phosphorylation of mitochondria. In mice models, mitoquinone was shown to diminish the content of macrophages, reduce cell proliferation in atherosclerotic plaques, and constrain multiple features of metabolic syndrome [91,92,93]. Edaravone is another oxygen radical scavenger and an inhibitor of lipid peroxidation with potent antioxidant effects. Edaravone has been shown to reduce ischemia and reperfusion-associated vascular endothelial cell injury, diminishing neuronal death, brain edema, and associated neurological deficits [94]. In a large meta-analysis, antioxidant treatments (such as vitamin C and selenium) showed benefit in vitro and in animal models of CVD but failed to improve outcomes in a human context. Concentrations of antioxidant agents used in in vitro studies may have been much higher than possible oral ingestion in humans. Therefore, better antioxidant strategies need to be developed [95].

In addition, activation of NADPH oxidases promotes the production of ROS from other sources. Therefore, NADPH is recognized as another therapeutic target. NADPH oxidase (Nox) inhibitors have been developed and were shown to be effective in atherosclerosis in mice models [96].

Treatment of diabetic animals with superoxide dismutase/catalase mimics can prevent the oxidative inactivation of aortic prostacyclin synthase caused by diabetes [97]. Transgenic antioxidant enzyme expression or a combination of antioxidant compounds to inhibit hyperglycemia-induced ROS production in diabetic mice can prevent the development of experimental diabetic neuropathy, cardiomyopathy, nephropathy, and retinopathy [98,99,100,101,102]. Thus, preventive treatment of excessive superoxide caused by diabetes could be a future target for the prevention of diabetes complications [41].

## 7. Conclusions

CVDs remain a leading cause of morbidity and mortality. Particularly in the western world, diabetes, smoking, lack of regular exercise, and hypertension lead to CVD. To date, a growing amount of evidence has demonstrated that the chronic CVD process is related to inflammation and oxidative stress, which involves many cells, including their interactions, such as dendritic cells, platelets, eosinophils, and adipocytes. Furthermore, factors promoting inflammation such as the complement system are central to this process. Chronic low-grade inflammation in metabolic disorders, such as obesity and insulin resistance, is associated with an excessive production of ROS, resulting in endothelial cell dysfunction and release of further pro-inflammatory cytokines, which ultimately aggravates the development of ROS-mediated inflammation and consecutively cardiovascular diseases. Thus, understanding the relationship between oxidative stress, inflammation, and cardiovascular diseases will offer ways to control inflammation and oxidative stress, and is a promising direction for developing treatment strategies of affected patients.

## Data Availability

Not applicable.
